# A Portable Chemotaxis Platform for Short and Long Term Analysis

**DOI:** 10.1371/journal.pone.0044995

**Published:** 2012-09-17

**Authors:** Chenjie Xu, Yuk Kee C. Poh, Isaac Roes, Eoin D. O'Cearbhaill, Mads Emil Matthiesen, Luye Mu, Seung Yun Yang, David Miranda-Nieves, Daniel Irimia, Jeffrey M. Karp

**Affiliations:** 1 Division of Biomedical Engineering, Department of Medicine, Center for Regenerative Therapeutics, Brigham and Women's Hospital, Harvard Medical School, Harvard Stem Cell Institute, Harvard-MIT, Division of Health Sciences and Technology, Cambridge, Massachusetts, United States of America; 2 BioMEMS Resource Center, Center for Engineering in Medicine and Surgical Services, Massachusetts General Hospital, Shriners Hospital for Children, and Harvard Medical School, Boston, Massachusetts, United States of America; 3 Division of Bioengineering, School of Chemical & Biomedical Engineering, Nanyang Technological University, Singapore; Stanford University, United States of America

## Abstract

Flow-based microfluidic systems have been widely utilized for cell migration studies given their ability to generate versatile and precisely defined chemical gradients and to permit direct visualization of migrating cells. Nonetheless, the general need for bulky peripherals such as mechanical pumps and tubing and the complicated setup procedures significantly limit the widespread use of these microfluidic systems for cell migration studies. Here we present a simple method to power microfluidic devices for chemotaxis assays using the commercially available ALZET® osmotic pumps. Specifically, we developed a standalone chemotaxis platform that has the same footprint as a multiwell plate and can generate well-defined, stable chemical gradients continuously for up to 7 days. Using this platform, we validated the short-term (24 hours) and long-term (72 hours) concentration dependent PDGF-BB chemotaxis response of human bone marrow derived mesenchymal stem cells.

## Introduction

While cell migration is critical for embryogenesis, homeostasis, and tissue regeneration, a simple, robust, and accessible *in vitro* assay for the study of chemotaxis that can generate long-term stable linear gradients from nearly any agent has remained elusive. Flow-based microfluidic gradient generators are useful to overcome many key limitations in traditional chemotaxis assays by providing exquisite control over chemotactic gradient profiles and permitting direct visualization and quantification of cell migration [1], [2], [3]. Gradient generating devices typically utilize diffusive mixing of parallel flowing streams of different concentrations to instantly generate a chemical gradient in the direction perpendicular to the flow [4], [5]. Due to the use of continuous fluid flow, these flow-based gradient generators maintain stable and robust gradients of chemoattractants for both short-term and long-term studies, which are critical for the study of chemotaxis of slow migrating cells, yet are not possible with traditional migration assays [6].

However, despite substantial improvements made by flow-based microfluidic gradient generators, traditional chemotaxis assays (e.g. modified Boyden chamber) remain the method of choice for studying cell migration due to their simplicity [3]. Flow-based systems are relatively complex to utilize (compared to placing transwell inserts in a multiwell plate) and often require bulky equipment such as external electrical or pneumatic pumps, tubing, and fittings.[2] Assembling these components is laborious and they cannot be simply integrated into common cell culture procedures [7]. This becomes particularly problematic for long-term chemotaxis studies where the experimental setup must be maintained under standard cell culture conditions for days (i.e. at 37°C and 5% CO_2_). Recent studies by Park et al have used small PDMS osmotic pumps, where the osmotic flow was driven by the transport of buffer solution from the microfluidic device into a bath of poly(ethylene glycol) solution [8]. However, for long-term studies (>12 hours), this system is impractical as it is limited by the small capacity of the osmotic pump (120 µL) which only lasts for several hours at the rate of 20 µL/hr. Furthermore, the need to fabricate osmotic pumps discourages the routine use of this system.

To enable general use of flow-based gradient generators in chemotaxis studies, the ideal platform should be easy to use and be portable. The latter is often overlooked in designing laboratory devices, but is critical to permit users to readily transfer platforms between existing lab equipments (i.e. cell culture hood, incubator, and microscope) [2]. Universal chemotaxis platforms should also work for a wide range of chemoattractants (e.g. small molecules, peptides, and protein based growth factors). The shape and steepness of the gradients should be easily controlled, and the gradients should rapidly stabilize and remain constant for at least 24 hours to permit the study of both slow and fast migrating cell types.

Here, we have developed a standalone chemotaxis platform that has the same footprint as a multiwell plate and can generate well-defined, stable chemical gradients for up to 7 days. We used commercially available osmotic pumps (ALZET® Osmotic Pump) to drive fluid flow in a microfluidic gradient generator [5]. ALZET® pumps were originally designed for continuous drug delivery in small animals; therefore, these pumps are compact (size of a micro-centrifuge tube), battery-free, and operate at a constant slow flow rate for days. This permits us to strip away all the bulky peripherals and power connections that are normally required in existing flow-based chemotaxis systems. Moreover, unstable chemoattractants inside the pump can easily be replaced by withdrawing old medium and infusing fresh medium into the pump maintaining their activity during long-term studies. For versatile control over the chemical gradients we used a universal microfluidic gradient generator [5]. Using mathematical modeling software, we could accurately predict the resultant gradient profiles given the identity of the chemoattractants. Using this platform, we validated the assay over the short and long term by examining the chemotactic response of human bone marrow derived Mesenchymal Stem Cells (MSCs) under a gradient of Platelet-Derived Growth Factor BB (PDGF-BB) for 24 hours and 72 hours respectively [9]. Given its simplicity and ability to generate long-term stable gradients, we believe combining microfluidic gradient generators and ALZET® osmotic pumps has the potential to become a universal routine assay for the study of chemotaxis.

## Results

**Figure 1:**
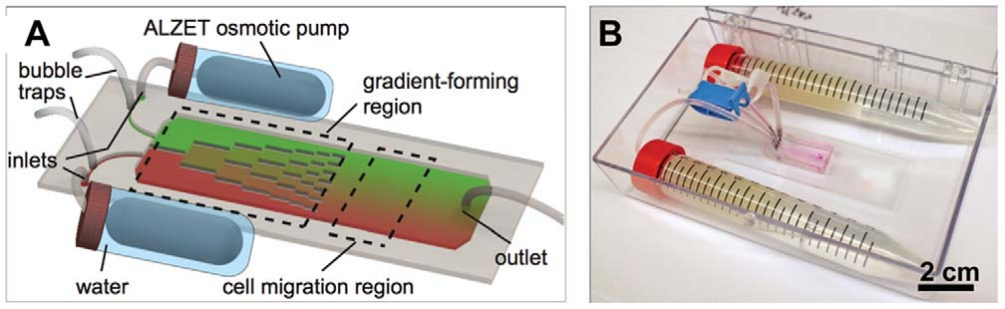
Osmotic pump powered microfluidic platform. (**A**) Schematic (not to scale) of the chemotaxis platform. Two ALZET® osmotic pumps are connected to a microfluidic gradient generator through flexible tubing. Gradient profile is controlled by the pump speed and the arrangement of the dividers within the gradient-forming region. (**B**) An image of the chemotaxis platform contained within a small plastic box.

The chemotaxis platform comprises of a microfluidic gradient generator and two osmotic pumps (ALZET®, Durect Corp., Cupertino, CA). The microfluidic device is adapted from our previous design, containing three components: a pair of inlets, a gradient-forming region, and a cell migration region (Figure 1A) [5]. This gradient generator enables creation of a stable gradient of any profile (e.g. linear, power, exponential) by modulating the inter-diffusion between two adjacent streams using multiple parallel dividers along the direction of flow. The arrangement of these dividers determines the resultant gradient profile. In the system presented here, solutions from the two inlets flow through the gradient-forming region and exit into the cell migration region, where a linear gradient is formed. The microfluidic device was connected to two ALZET® pumps that were immersed in phosphate buffered saline (PBS) inside two conical tubes to keep the pumps hydrated. The flow rate reaches the nominal value when pumps are hydrated at 37°C. Bubble traps were added along the inlet channels to prevent bubbles from entering the microfluidic device that could destroy the gradient and damage the adherent cells (Figure 1A). The assembly was placed inside a small transparent plastic box (size of a multiwell plate) (Figure 1B) that can be conveniently stored in a standard incubator and transferred between a culture hood and a microscope.

**Figure 2:**
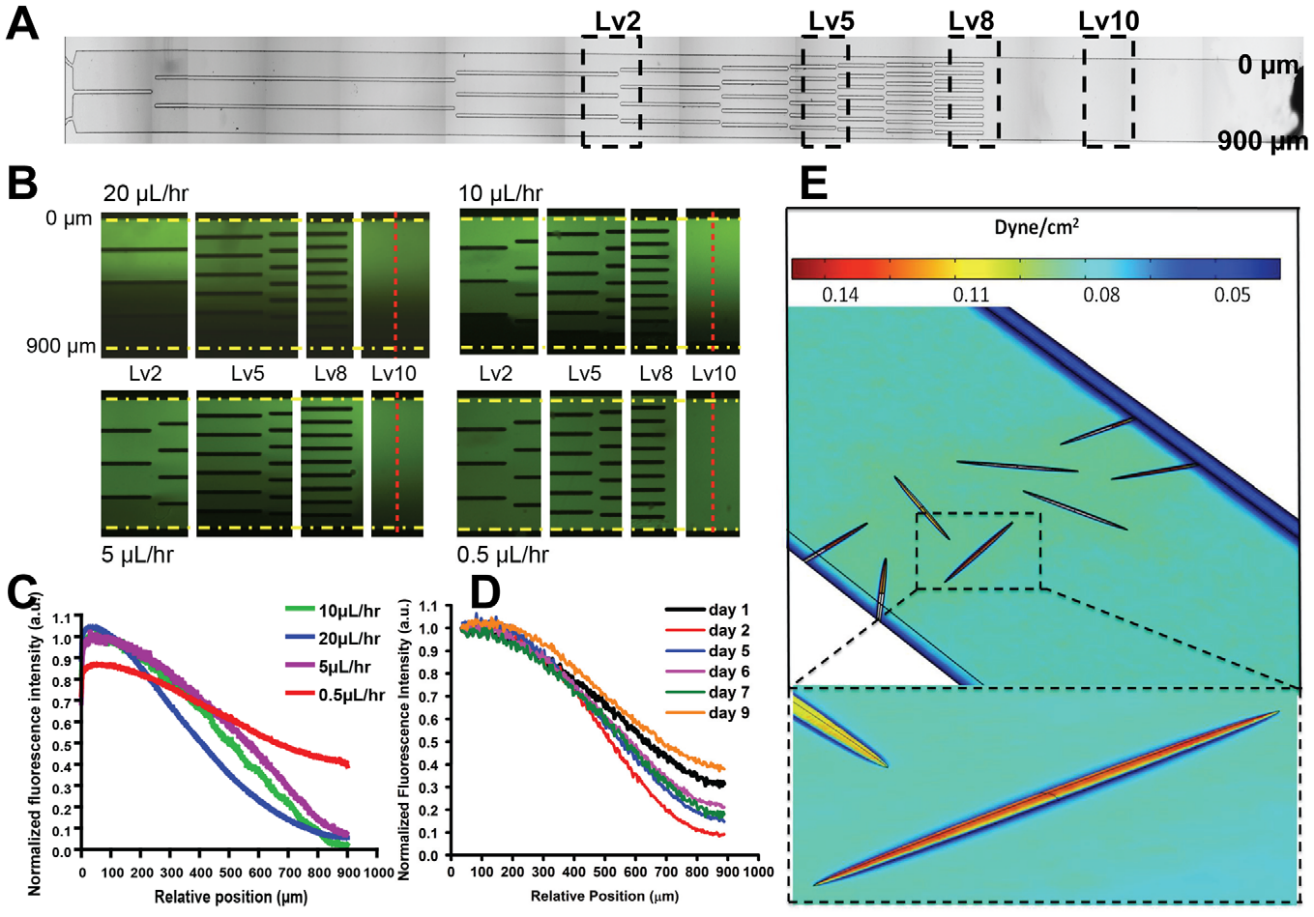
Gradient evolution inside the microfluidic gradient generator. (**A**) A macroscopic image of the gradient generator. (**B–C**) Gradient evolution inside the microfluidic gradient generator at different pumping rates powered by mechanical pump: (**B**) Visualization of the gradient at level 2 (Lv2), 5 (Lv5), 8 (Lv8), and 10 (Lv10)), as denoted by the dashed boxes. Fluorescence images were captured within each zone 2 hours after starting the pump. Fluorescence intensity was measured in the middle of the cell migration region denoted by the red dashed lines. Yellow dashed lines denote the upper and lower boundaries of the microchannel. (**C**) Normalized fluorescence intensity of the fluorescein gradients along the cell migration channel (red dashed line in b) at different pumping rates. (**D**) Fluorescein gradient evolution across the cell migration region (Lv10) inside the microfluidic gradient generator powered by ALZET® osmotic pumps (5 µL/hr) throughout a 9-day period. Normalized by taking the fluorescent intensity at 0 µm as 1. (**E**) Shear stress within the cell migration region modeled using COMSOL. Inset at bottom: a model cell (height 1.5 µm), experiences shear stresses in the range of 0.03–0.14 dynes/cm^2^.

A critical consideration was the selection of the pumping rate. Extremely slow flow rates will not generate a stable gradient, whereas high flow rates may impose excessive shear stress on the adherent cells. To determine the optimal pumping rate, we perfused fluorescein sodium salt solution (fluorescein) and PBS into the two inlets using a mechanical syringe pump under four flow rates (20, 10, 5, and 0.5 µL/hr). As shown in Figure 2B & 2C, a fluorescein gradient was generated within the cell migration region (denoted as Lv 10 in Figure 2A) under all flow rates. Gradients generated under 5, 10 and 20 µL/hr had the highest linearity and exhibited the largest concentration range. At 0.5 µL/hr, the flow was so slow that sufficient mixing had taken place near the upper and lower boundaries of the microchannel, resulting in a decreased concentration range of the gradient. We chose a pumping rate of 5 µL/hr to maximize the gradient range while minimizing shear stress induced by the flow and consumption of chemoattractants. By powering the microfluidic device with two ALZET® osmotic pumps rated at 5 µL/hr, we achieved a similar fluorescein gradient to those generated by the mechanical syringe pump at the same pumping rate (Figure S1).

To show that gradients generated can reliably be predicted through mathematical modeling, we modeled the gradient profiles of three different-sized molecules (376 Da fluorescein sodium salt, 4 kDa and 70 kDa fluorescein isothiocyanate–dextran (FITC-dextran)) and compared them to experimental observations (Figure S2). Quantification of the fluorescence intensity in the cell migration region (Lv10) shows that our experimental profile matches closely with our theoretical profile (<5% variability).

Chemotaxis *in vivo* typically occurs on the order of days during several biological processes (e.g. embryonic development, stem cell differentiation, wound healing, and growth of axon) [10], [11], [12]. Flow-based chemotaxis assays offer excellent control over the chemotactic gradient but they are difficult to be maintained inside the standard cell incubator with typical culturing conditions (37°C, 5% CO_2_, 100% humidity) for days, owing to the need for bulky peripheral equipments. On the other hand, traditional transwell assays can be conveniently handled in the form of multiwell plates but their control over the gradient is poor and the stability of the gradient only lasts for a few hours. Here, we show that by powering our chemotaxis platform with two ALZET® osmotic pumps, our chemotaxis platform can achieve both exquisite control and long-term stability over the gradient. As shown in Figure 2D, our platform is able to maintain a stable, linear gradient for up to 7 days, which is the rated lifetime of the ALZET® osmotic pump. At day 9, the quality of the gradient had diminished. Due to the low flow rate used in our platform, less than 1.7 mL of chemoattractants was consumed over the course of 7 days.

One of the possible consequences of applying flow is that the fluid shear stress inside the microfluidic channel may negatively impact cell viability and proliferation [13], [14]. We performed theoretical modeling to predict the wall shear stress in the cell migration region of the device (Figure 2E). Modeling results showed that a flow rate of 10 µL/hr (i.e. 5 µL/hr at each of the two inlets) would generate 0.14 dynes/cm^2^ and 0.03 dynes/cm^2^ on the top and bottom of a cell, respectively. This level of shear stress has been shown to have minimal impact on cell phenotype [14]. We confirmed this by examining cell viability and morphology of MSCs under flow at 10 µL/hr for three days (Figure S3). At the end of three days, we observed a slight proliferation of MSCs as the number of cells in the cell migration region increased from 132 (0 hour) to 152 (72 hours) (Figure S3A), and we did not observe any significant changes in cell morphology (Figure S3B). These results suggest that the platform may be suitable for performing long-term chemotaxis assays (on the order of days), where continuous exposure to very low shear stress (0.03–0.14 dynes/cm^2^) would have limited effects on cell viability and morphology.

**Figure 3:**
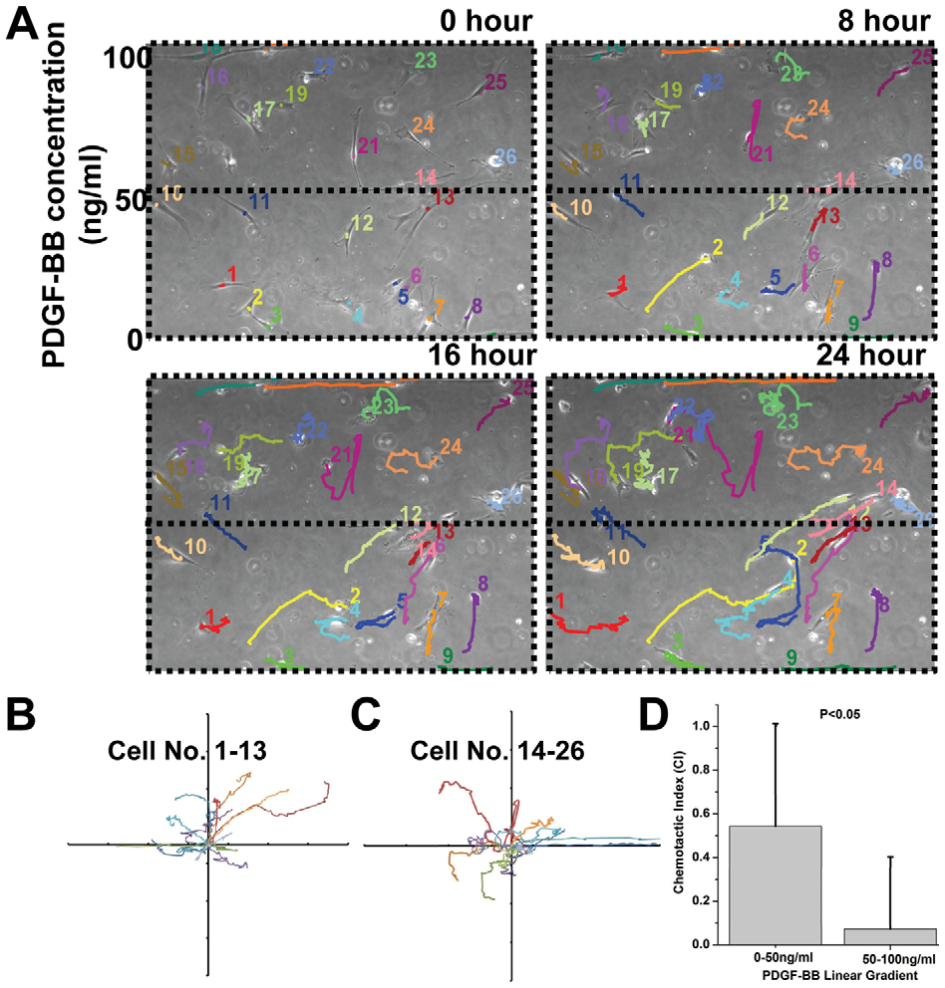
Microfluidic platform for short-term chemotaxis assay. (**A**) Time-lapse images of MSCs migration under a PDGF-BB gradient for 24 hours. Images were taken every 15 minutes and individual color-coded cell tracks were assembled after 0, 8, 16, and 24 hours. A movie clip of the 24-hour cell migration data is available (Mov. S1). (**B, C**) Migration traces of cells initially seeded in the lower PDGF-BB concentration region (cell no. 1-13) and in the higher PDGF-BB concentration region (cell no. 14-26), respectively. These cell traces (**B**) indicate that cells in the bottom half of the channel (0–50 ng/mL of PDGF-BB) exhibited directed migration, whereas (**C**) cells in the top half of the channel (50–100 ng/mL of PDGF-BB) exhibited random motion. Axes are in the units of 200 microns. (**D**) Chemotactic index, CI of MSCs in 0–50 ng/ml and 50–100 ng/ml PDGF-BB regions. Statistical significance was determined by Student's *t*-test comparing cells in the bottom and top parts of the channel (*p<0.05).

Using this chemotaxis platform, we examined the short-term and long-term effects of PDGF-BB gradient on MSCs migration. PDGF-BB is a well-studied chemotactic cue to many mammalian cell types, including MSCs [9], [15]. Currently, the chemotactic response of MSCs in a PDGF-BB gradient is mainly studied using modified Boyden Chambers, which only provides a temporal PDGF-BB gradient for a few hours. Results achieved using Boyden Chambers do not provide any information about the real-time response of MSCs towards a stable PDGF-BB gradient or the long term effect of the gradient over cell migration. Here, we established a linear PDGF-BB gradient (0 to 100 ng/mL) in the cell migration region and observed the migratory response of MSCs for 24 hours using live-cell imaging (Figure 3 & Mov. S1). We also modeled the PDGF-BB gradient using COMSOL to verify the profile shape (Figure S4). As shown in Figure 3, MSCs that were initially exposed to the lower concentrations of the PDGF-BB gradient (0–50 ng/ml, cell no. 1-13 in Figure 3A & 3B) exhibited directed migration towards the higher concentrations of the PDGF-BB gradient (50–100 ng/mL, upper half of the channel). The longest displacement of MSCs along the direction of gradient was 296 µm (∼12 µm/hour) (cell No. 6, Figure 3A & 3B). Conversely, the majority of MSCs that were initially exposed to the higher concentrations of the PDGF-BB gradient (cell no. 14-26 in Figure 3A & 3C) showed a random migration pattern (i.e. chemokinesis). The different migration behavior of cells between 0–50 ng/ml region and 50–100 ng/ml region was confirmed through examining chemotactic index (CI) [16]. The CI of MSCs in the 0–50 ng/ml region was much higher than that of MSCs in 50–100 ng/ml region (Figure 3D), which agrees with previous studies that have shown that MSCs exhibit a chemotactic response towards PDGF-BB and the maximal response is typically observed in the range of 10–50 ng/mL [17].

**Figure 4:**
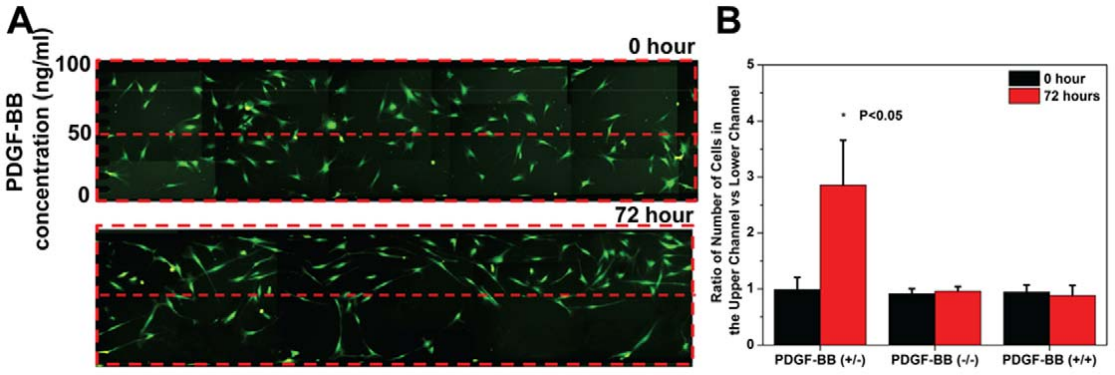
Microfluidic platform for long-term chemotaxis assay. (**A**) Long-term migration of MSCs (labeled with CFSE/Calcein AM) within a PDGF-BB gradient (0–100 ng/ml). The total number of cells present within the cell migration region was 105 at 0 hour and 107 at 72 hours. Limited by the visualization area of microscope, fluorescent images of adjacent areas were taken individually and spliced together. (**B**) Cell distribution within the cell migration region in the presence (0–100 ng/mL PDGF-BB) or absence (0-0 ng/mL or 100-100 ng/mL PDGF-BB) of a chemotactic gradient. Data from Figure 4A, S3, and S5 were represented as ratios of number of cells present in the upper half of the channel to that in the lower half of the channel. Results are means ± STD for n = 3. Statistical significance was determined by Student's *t*-test comparing results in the presence of a gradient from 0 and 72 hours (*p<0.05).

One of the unmet needs in chemotaxis studies is that it is very difficult to perform long-term assays (on the order of days). To validate the microfluidic platform for long-term chemotaxis analysis, we examined the effects of a PDGF-BB gradient (0–100 ng/mL) on MSCs migration for 72 hours (Figure 4 and Figure S7). In one typical example (Figure 4A), 72 hours after introducing the gradient, the number of cells in the region with lower PDGF-BB concentrations (0–50 ng/mL, bottom region) decreased significantly from 50 to 25, whereas the number of cells in the region with higher PDGF-BB concentrations (50–100 ng/mL, top region) increased from 55 to 82. The accumulated data from three independent experiments revealed that the ratio of the number of MSCs in the top region vs. the bottom region significantly increased from 0.98±0.21 to 2.85±0.8, 72 hours after the introduction of the gradient (Figure 4B), indicating that MSCs underwent a directed migration in response to the PDGF-BB gradient. In the absence of a PDGF-BB gradient (i.e. 0–0 ng/mL (−/−) or 100-100 ng/mL (+/+) of PDGF-BB), we did not observe a significant change in cell distribution (Figure 4B and Figure S2 & S5), confirming our observation in Figure 4A is a result of long-term directed migration in the presence of a PDGF-BB gradient. The total number of MSCs in the channel did not change significantly before and 72 hours after introducing the gradient indicating that the flow stream did not detach cells, and calcein AM imaging indicated that the viability of MSCs was minimally impacted (Figure S6).

The precise control over the chemotactic gradients for more than 3 days described here is a unique feature among chemotaxis assays. Traditional assays such as modified Boyden chambers, Dunn chambers, and under-agarose assays exhibit gradients that quickly deteriorate (in less than 24 hours) and they offer limited control over the gradient profiles [18]. Flow-based microfluidic gradient generators facilitate long-term stable chemotactic gradients with excellent control over the gradient profiles, but require bulky peripherals [19]. Furthermore, non-trivial steps in maintaining the existing gradient generators under culture conditions for days present significant challenges for wide adoption. The long-term stability of the gradients enables studies of slowly migrating adherent cells including fibroblasts, cancer cells, neurons, and stem cells.

## Conclusion

In summary, we have designed a simple, robust, and accessible system for the study of chemotaxis experiments lasting several days. The device includes a microfluidic chamber and two commercially available ALZET® osmotic pumps, is easy to assemble, avoids the use of bulky peripheral devices, has a small footprint, requires minimal maintenance, and is capable of generating stable gradients that can persist for 7 days. Importantly, low cost microfluidic devices for academic research can be fabricated through government sponsored foundries (e.g. http://www.stanford.edu/group/foundry/). The assembled device was used to show the short-term and long-term directional migration of MSCs under a PDGF-BB gradient. This simple portable microfluidic platform has potential to become widely adopted for experiments requiring stable gradients, and may find use in non-laboratory settings.

## Materials and Methods

### Mesenchymal stem cell culture and characterization

Primary human MSCs were obtained from the Texas A&M Health Science Center, College of Medicine, Institute for Regenerative Medicine at Scott & White Hospital which has a grant from NCRR of the NIH, Grant #P40RR017447. MSCs were derived from healthy consenting donors and thoroughly characterized as previously described [20]. MSCs were maintained in a-MEM expansion media (Invitrogen) supplemented with 10% Fetal Bovine Serum (Premium Select, Atlanta Biologicals), 1% (v/v) L-Glutamine (Invitrogen), and 1% penicillin∶streptomycin solution (Invitrogen). Cells were cultured to 70–80% confluence before passaging. All experiments were performed using MSCs at passage number 3–6 where cells expressed high levels of MSC markers CD90 and CD29 (>99% cells), and did not express hematopoietic markers CD34 or CD45 (0% of cells) as observed from flow cytometry analysis. Prior to cell experiments, MSCs were detached with 1× Trypsin (Gibco) and filtered with 40 µm Nylon Mesh (Fisher Scientific).

### Fabrication of gradient generator

Standard microfabrication technology was used to fabricate 10 µm-wide dividers in 900 µm-wide and 40 µm-high channels in poly-(dimethylsiloxane) (PDMS, Sylgard 184; Dow Corning, Midland, MI) on glass. Briefly, a 40 µm layer of SU8 (Microchem, Newton,MA) was spun on a silicon wafer and photo-patterned according to manufacturer's instructions and using a Mylar mask (Fineline Imaging, Colorado Springs, CO). PDMS was prepared according to the manufacturer's instructions and cast over the developed photoresist mold to create complementary microchannels in PDMS. Through holes, defining the inlets and outlets, were punched using a flat bottomed 25-gauge needle. The bonding surfaces of the PDMS and a regular glass slide (1×3 inch; Fisher Scientific) were treated with oxygen plasma (150 mTorr, 50 W, 20 s) produced in the parallel plate plasma asher (March Inc., Concord, CA). Before use, devices were sterilized with 70% ethanol, coated with 5 µg/mL fibronectin for 45 min, washed with PBS, and equilibrated with full media.

### Establishment of a fluorescence gradient profile with a mechanical pump and analysis

An automatic syringe pump (Harvard Apparatus PHD 2000) was used to study the effect of flow rate on gradient generation. Prior to use, the microfluidic channel was pre-wetted with PBS for at least 30 minutes. One syringe was loaded with 0.1% Fluorescein sodium salt (FITC) (Aldrich) dye in PBS solution and the other syringe with pure PBS. Both syringes were connected to the microfluidic channel inlets and each syringe was pumped at flow rates of 20, 10, 5, and 0.5 µL/hr, which correspond to flow rates of 40, 20, 10, and 1 µL/hr and flow velocities of 352, 176, 88, and 8.8 µm/s in the cell migration region. Gradients were permitted sufficient time to establish and stabilize. The gradient was imaged using an inverted fluorescence microscope (TE2000, Nikon, Japan) equipped with a CCD camera.

### Establishment of a fluorescence gradient profile with ALZET® osmotic pump and analysis

Fluorescence gradient generation was confirmed using ALZET® osmotic pumps with a flow rate of 5 µL/hr. The gradient generator was pre-wetted with PBS at least for 30 min. Pumps were filled with the same solutions as above (FITC and PBS) and incubated in sterile PBS. Then the pumps and bubble trap were connected into the inlets of the devices and the gradient was allowed to stabilize for 1 hour. The gradient was imaged every 24 hours thereafter on an inverted fluorescence microscope (TE2000, Nikon) equipped with a CCD camera and temperature-maintaining system. The images were analyzed by ImageJ.

### Preparation of ALZET® osmotic pump for the study of cell migration

ALZET® osmotic pumps were prepared 24 hours ahead of experiment. The media containing 10% FBS or PDGF-BB (100 ng/ml in complete media) was pre-equilibrated within a cell incubator for 6 hours to allow sufficient gas exchange. The pre-equilibrated media was injected into ALZET® osmotic pumps. Finally, each media containing ALZET® osmotic pump was placed inside a 15 ml-conical tube filled with water and connected to 8 cm gas-permeable silicon tubing (Versili® SPX-50, Saint-Gobain Performance Plastics Corp.) that ran through the cap of the conical tubing. The final pump systems were placed in an incubator overnight to remove air bubbles that were introduced during the media loading process.

### FBS or PDGF-BB gradient generation with ALZET® osmotic pump

The devices were disinfected with 70% ethanol, coated with 5 µg/mL fibronectin for 45 min, washed with PBS, and equilibrated with full media for 1 hour. To facilitate cell tracking, MSCs were stained with 5 µg/mL CFSE (Molecular Probes) based on the manufacturer's standard protocol. The CFSE stained MSCs were detached and seeded through the outlet with 5 µL of cell suspension at a density of 1.5×10^6^ cells/mL. Following 6 hours to permit cell attachment, the device was connected with previously prepared ALZET® osmotic pumps.

For the live-cell imaging in the PDGF-BB gradient, the device was imaged every 15 min on a microscope (TE2000, Nikon, Japan) equipped with a CCD camera and temperature-maintaining system. Orientation bias of cells was quantified by chemotactic index (CI) [16], which is defined as the displacement along the direction of the gradient divided by the total displacement.

For the long-term migration, the device was maintained in a standard cell incubator. Prior to imaging, 0.5–1 mL PBS was added to the top of the outlet to prevent air bubbles from entering the channel. At the end of the experiment, calcein AM staining was used to label the living cells. Introduction of calcein AM staining solutions within the microfluidic device is convenient and simple, as the negative pressure generated by the temperature drop from 37°C to room temperature allows backward suction through the outlet. Calcein AM staining solution was placed on the outlet and the device was removed from the incubator for 30 min to allow calcein AM solution to enter the channel. The device was then placed in the cell incubator for 1 hour to pump out the calcein AM solution prior to imaging.

### Computational evaluation of gradient profile and shear stress

Three-dimensional flow in the microfluidic channel was modeled using COMSOL Multiphysics 4.2 finite element method. Diffusion coefficients of three molecules used in the modeling are Fluorescein sodium salt (MW = 376.27; 3.3×10^−6^ cm^2^ s^−1^), Dextran-FITC (MW = 4000, 1.48×10^−6^ cm^2^ s^−1^), and Dextran-FITC (MW = 70000, 0.36×10^−6^ cm^2^ s^−1^), and PDGF-BB (MW = 24300, 1.29×10^−6^ cm^2^ s^−1^) [21].

Computational simulations were used to evaluate the wall shear stress distribution in the channel and identify the regions in which cells would experience minimal shear stress. Flow in the microfluidic device was assumed to be laminar flow of an incompressible Newtonian fluid. The physical properties (i.e. density and viscosity) of cell culture media were taken to be that of water at 37°C. The cell model used in the shear stress simulations was based on experimental observations [22] and the model described by Gaver and Kute [23]. The cell was taken to be a semi ellipsoid with semi-axes a = 130 µm (length), b = 5 µm (width) and c = 1.5 µm (height),.To model the environment of the microfluidic channel, two strands of four cells, randomly orientated, were spread across the bottom face of a cube the width and height of the real cell chamber. The inlet had laminar inflow at 10 µL/hr, no-slip boundary conditions and laminar outflow. The simulation was performed using a mesh with 1,236,479 elements over the computational domain (0.1044 mm^3^) with dramatically refined mesh elements around the model cells.

## Supporting Information

Figure S1 **Comparison of gradient profiles (measured along the red dashed lines in** **Figure 2B** **) generated at 5 µL/hr by a mechanical syringe pump vs ALZET® osmotic pumps.**
(TIF)

Figure S2 **Comparison of theoretical modeling and experimental result of gradients generated at a pumping rate of 5 µL/hr by ALZET® osmotic pumps.** We modeled and tested three different fluorescent molecules: (**A**) 376 Da fluorescein sodium salt, (**B**) 4 kDa FITC-dextran, and (**C**) 70 kDa FITC-dextran. Gradient profiles shown were measured within the cell migration region (Lv10).
(TIF)

Figure S3 (**A**) Tracking MSC response under flow for 3 days. MSCs labeled with CFSE dye were plated within the cell migration region of the device and subjected to a shear flow of media containing 10% FBS at 10 µL/hr for 3 days. 24 hours after seeding, the number of adherent cells was 132 (0 hour). 3 days later, the cell number increased to 152 (72 hours), indicating that cells were not compromised and exhibited a slow proliferation rate within the device under flow conditions. Limited by the visualization area of microscope, fluorescent images of adjacent areas were taken individually and spliced together. (**B**) Morphology characterization (area per cell, circularity index, and aspect ratio) of MSCs following exposure to shear flow for 3 days.
(TIF)

Figure S4 **Theoretical modeling of PDGF-BB gradient within the cell migration region at pumping speed of 5 µL/hr**
(TIF)

Figure S5 **Lack of migration of CFSE stained MSCs within PDGF-BB containing media (100 ng/ml) in both the upper and lower channels by 5 µL/hr osmotic pump.** (Total cell number was 131 at 0 hour and 142 at 72 hours). Limited by the visualization area of microscope, fluorescent images of adjacent areas were taken individually and spliced together.
(TIF)

Figure S6 **Total number of viable MSCs.** (Determined by Calcein AM staining in the channel at 0 and 72 hours under three conditions. Results are means ± STD for n = 3.)
(TIF)

Figure S7 **Tracking MSC response under flow for 4 days.** (**A**) MSCs labeled with CFSE dye were plated within the cell migration region of the device and subjected to a shear flow of media containing 10% FBS at 10 µL/hr for 4 days. Limited by the visualization area of the microscope, fluorescent images of adjacent areas were taken individually and spliced together. (**B**) Cell distribution within the cell migration region in the presence (i.e. 0–100 ng/mL PDGF-BB) of a chemotactic gradient for 4 days. Data were represented as ratios of number of cells present in the upper half of the channel to that in the lower half of the channel. Results are means ± STD for n = 3. Statistical analysis was performed by a one way ANOVA with Tukey's HSD post-hoc analysis for multiple comparisons, and p-values<0.05 were considered statistically significant, labeled with *.
(TIF)

Movie S1 **24-hour MSC migration under the PDGF-BB gradient of 100-0 ng/ml in the microfluidic device.**
(AVI)
