# Supplementary figures and images for: A Portable Chemotaxis Platform for Short and Long Term Analysis
Source: PLoS One. 2012 Sep 17;7(9):e44995. doi: 10.1371/journal.pone.0044995 (PMC3444504; doi:10.1371/journal.pone.0044995)

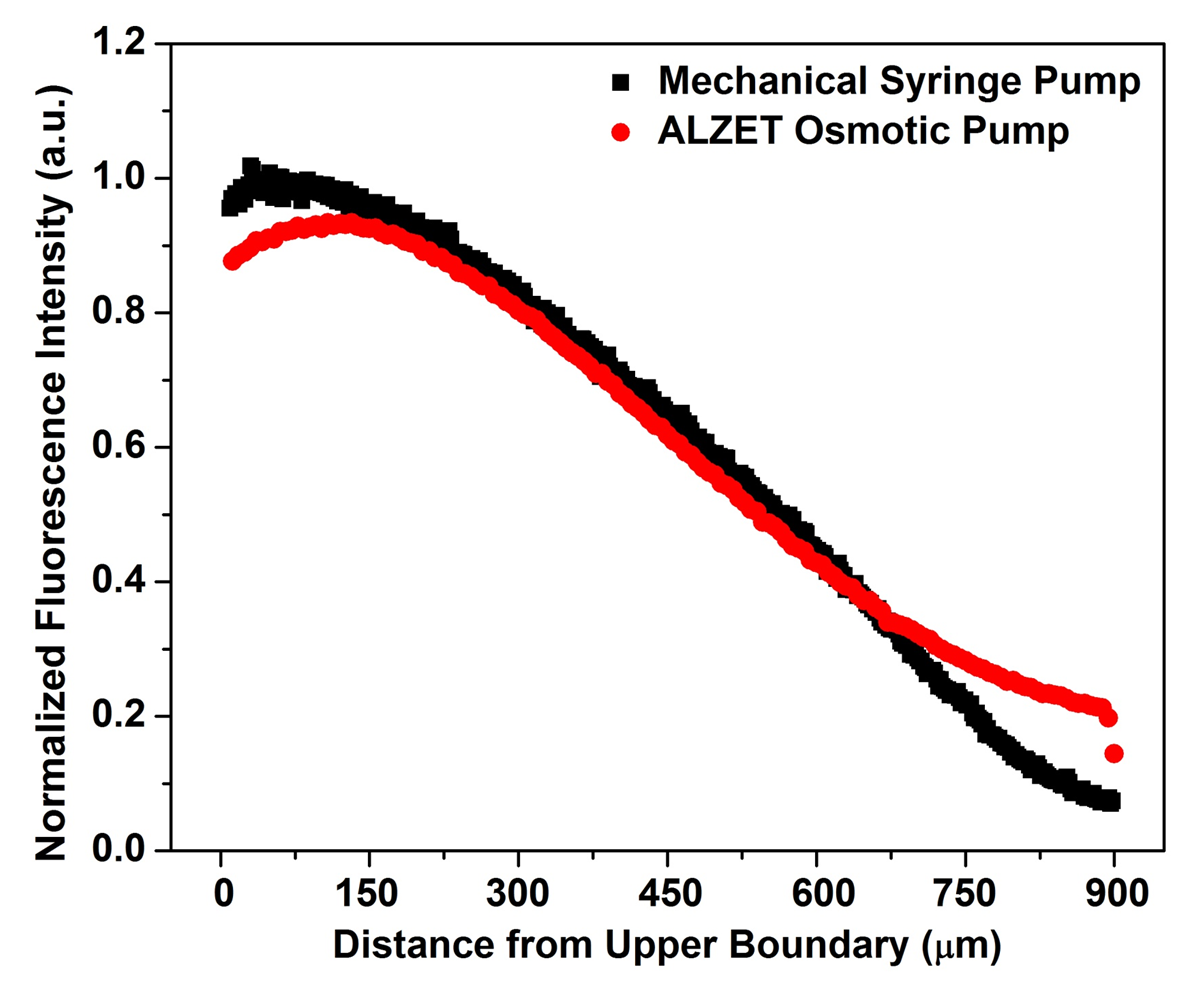

Supplement: Figure S1 — Comparison of gradient profiles (measured along the red dashed lines in Figure 2B ) generated at 5 µL/hr by a mechanical syringe pump vs ALZET® osmotic pumps. (TIF) [file pone.0044995.s001.tif]

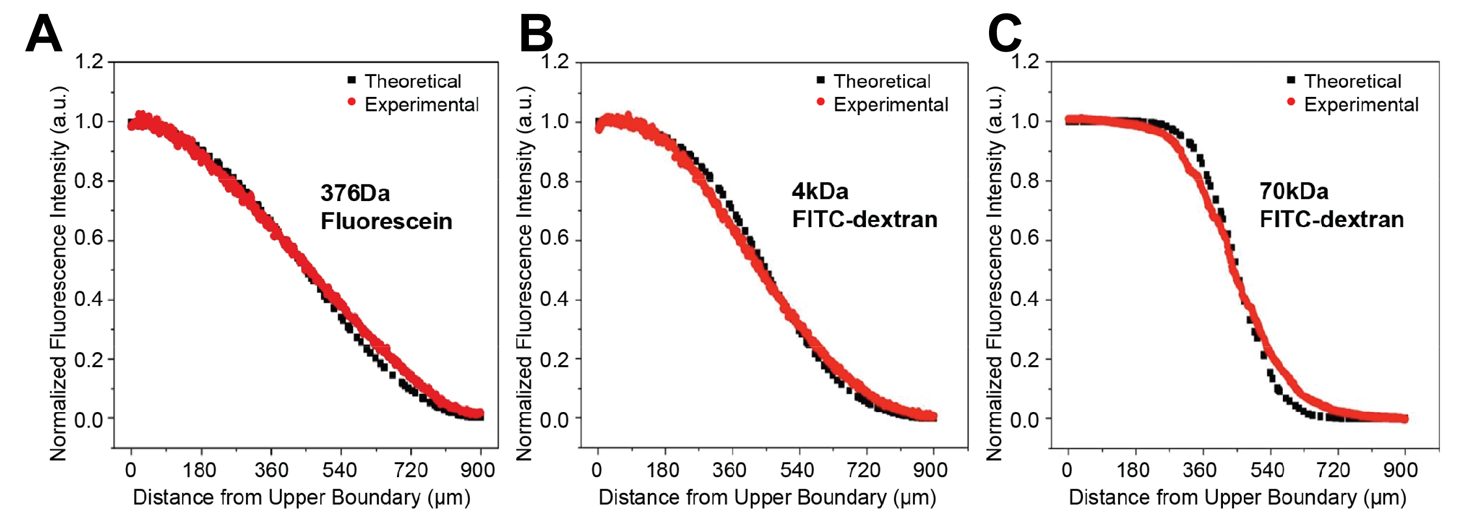

Supplement: Figure S2 — Comparison of theoretical modeling and experimental result of gradients generated at a pumping rate of 5 µL/hr by ALZET® osmotic pumps. We modeled and tested three different fluorescent molecules: (A) 376 Da fluorescein sodium salt, (B) 4 kDa FITC-dextran, and (C) 70 kDa FITC-dextran. Gradient profiles shown were measured within the cell migration region (Lv10). (TIF) [file pone.0044995.s002.tif]

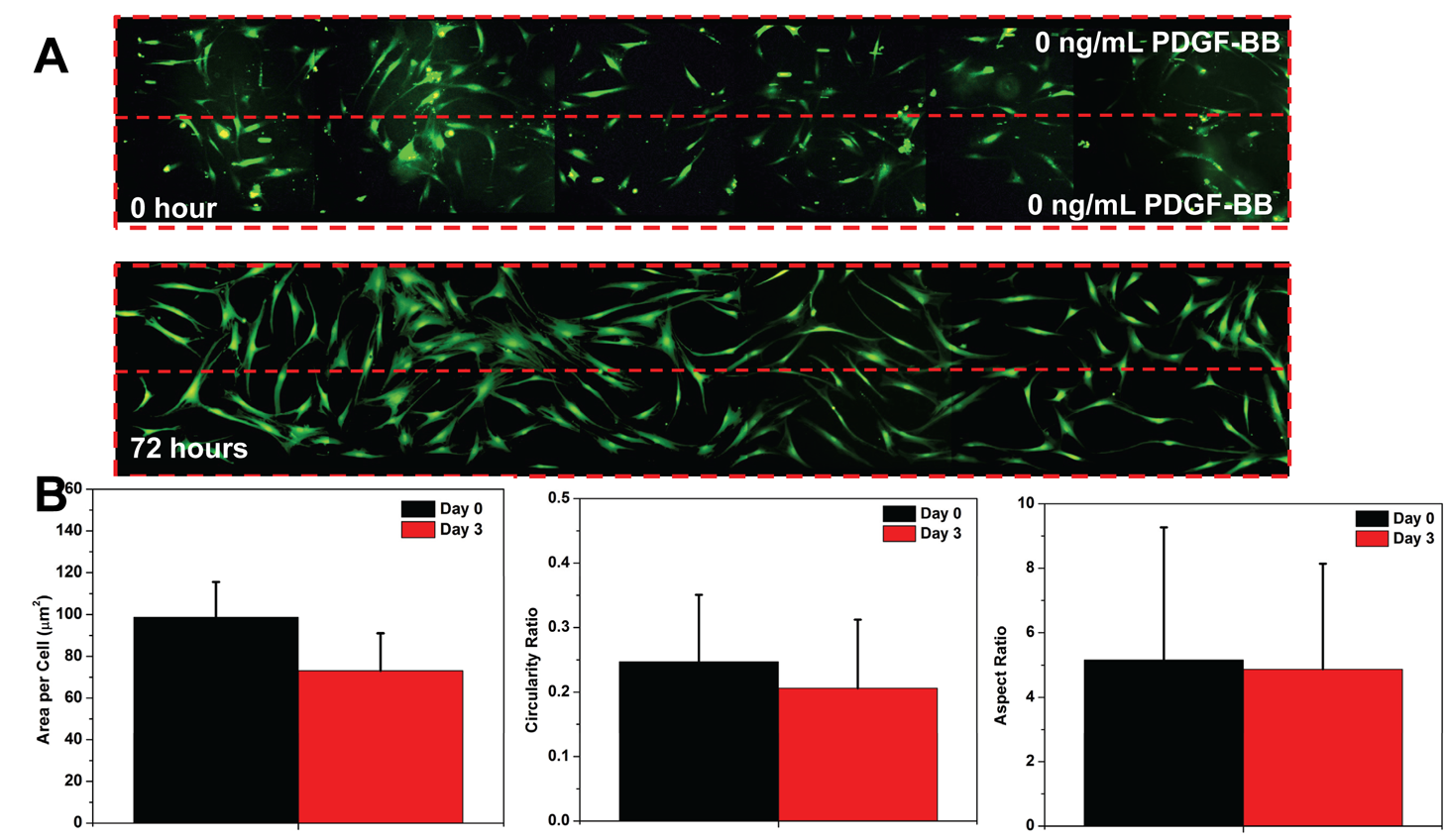

Supplement: Figure S3 — (A) Tracking MSC response under flow for 3 days. MSCs labeled with CFSE dye were plated within the cell migration region of the device and subjected to a shear flow of media containing 10% FBS at 10 µL/hr for 3 days. 24 hours after seeding, the number of adherent cells was 132 (0 hour). 3 days later, the cell number increased to 152 (72 hours), indicating that cells were not compromised and exhibited a slow proliferation rate within the device under flow conditions. Limited by the visualization area of microscope, fluorescent images of adjacent areas were taken individually and spliced together. (B) Morphology characterization (area per cell, circularity index, and aspect ratio) of MSCs following exposure to shear flow for 3 days. (TIF) [file pone.0044995.s003.tif]

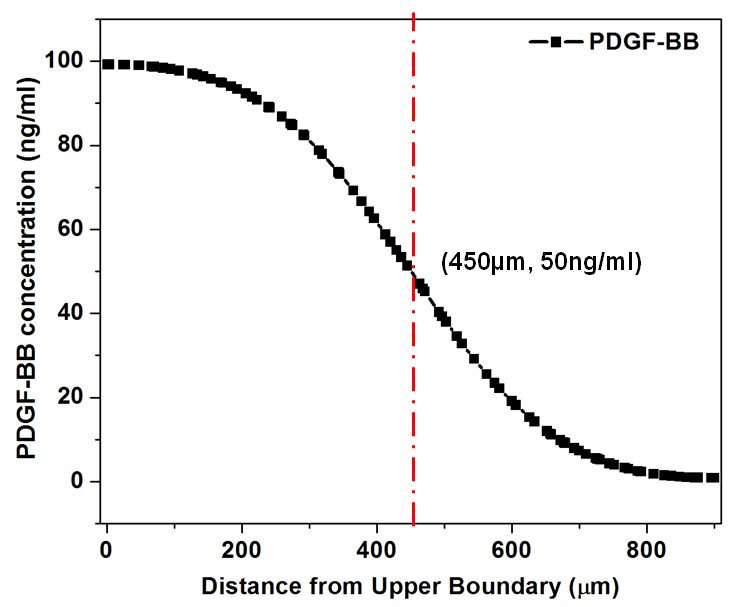

Supplement: Figure S4 — Theoretical modeling of PDGF-BB gradient within the cell migration region at pumping speed of 5 µL/hr (TIF) [file pone.0044995.s004.tif]

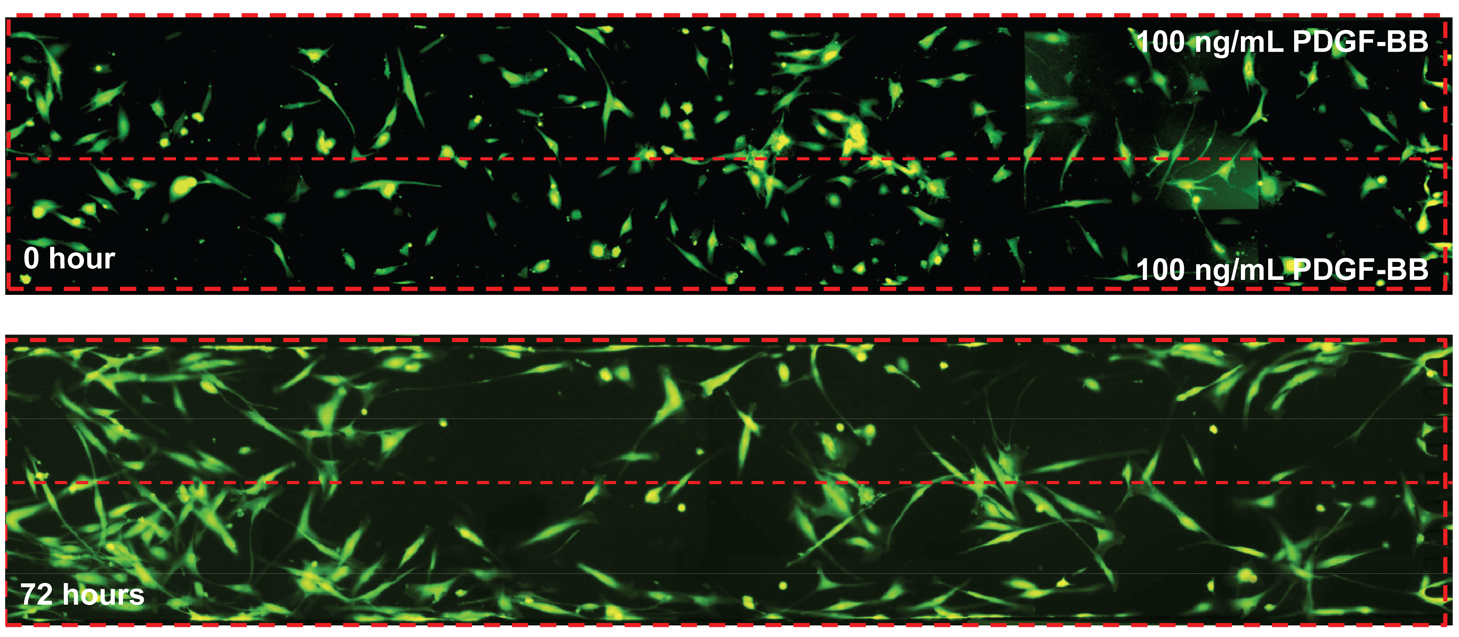

Supplement: Figure S5 — Lack of migration of CFSE stained MSCs within PDGF-BB containing media (100 ng/ml) in both the upper and lower channels by 5 µL/hr osmotic pump. (Total cell number was 131 at 0 hour and 142 at 72 hours). Limited by the visualization area of microscope, fluorescent images of adjacent areas were taken individually and spliced together. (TIF) [file pone.0044995.s005.tif]

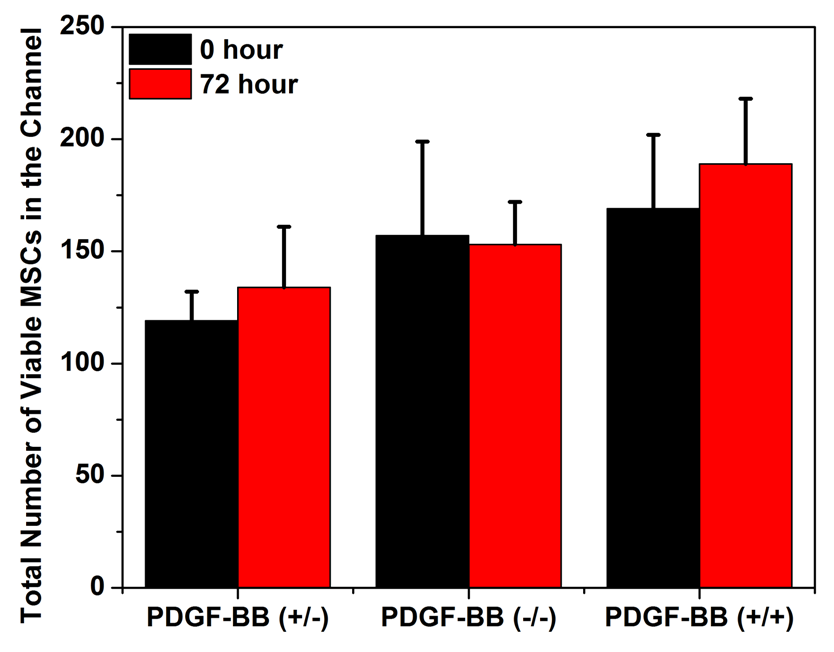

Supplement: Figure S6 — Total number of viable MSCs. (Determined by Calcein AM staining in the channel at 0 and 72 hours under three conditions. Results are means ± STD for n = 3.) (TIF) [file pone.0044995.s006.tif]

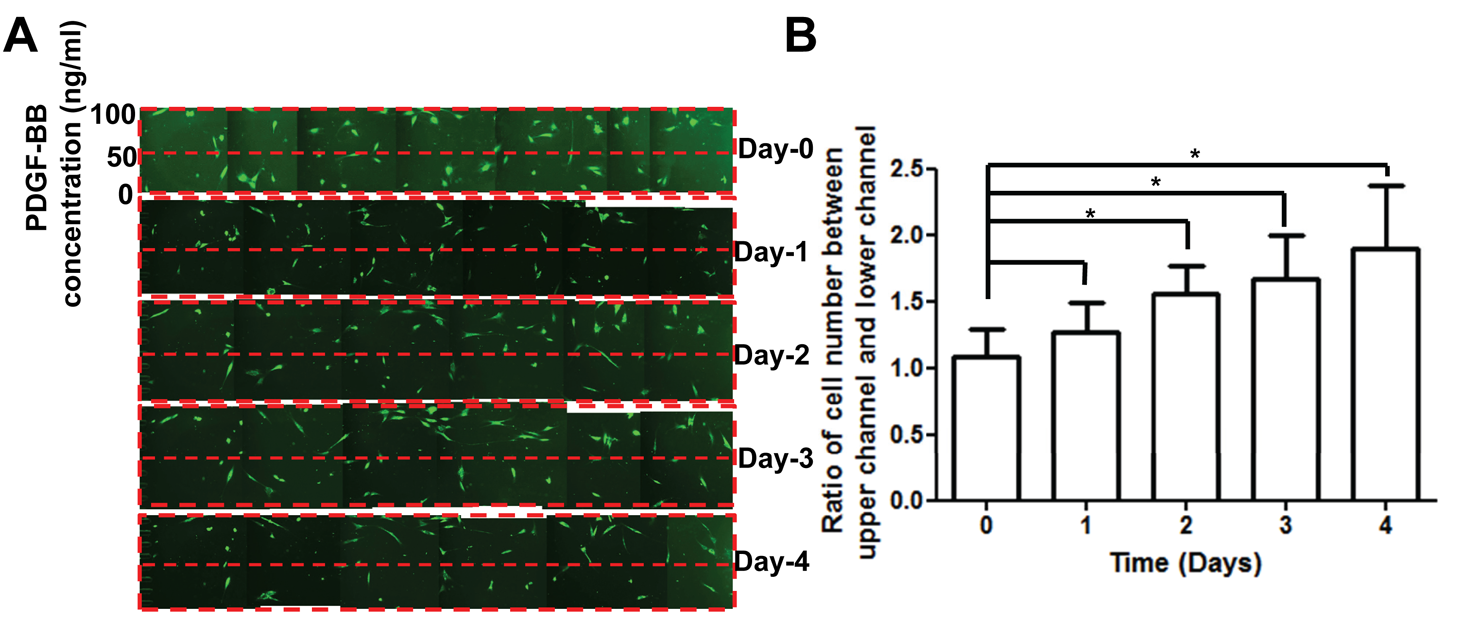

Supplement: Figure S7 — Tracking MSC response under flow for 4 days. (A) MSCs labeled with CFSE dye were plated within the cell migration region of the device and subjected to a shear flow of media containing 10% FBS at 10 µL/hr for 4 days. Limited by the visualization area of the microscope, fluorescent images of adjacent areas were taken individually and spliced together. (B) Cell distribution within the cell migration region in the presence (i.e. 0–100 ng/mL PDGF-BB) of a chemotactic gradient for 4 days. Data were represented as ratios of number of cells present in the upper half of the channel to that in the lower half of the channel. Results are means ± STD for n = 3. Statistical analysis was performed by a one way ANOVA with Tukey's HSD post-hoc analysis for multiple comparisons, and p-values<0.05 were considered statistically significant, labeled with *. (TIF) [file pone.0044995.s007.tif]
